# Supplementary material for: A pilot study on bio-banding in male youth ice hockey: Players’ perceptions and coaches’ selection preferences
Source: PLoS One. 2024 Aug 12;19(8):e0308676. doi: 10.1371/journal.pone.0308676 (PMC11318913; doi:10.1371/journal.pone.0308676)
Supplement: S1 File — (PDF) [file pone.0308676.s001.pdf]

**Supporting file S1**

# A pilot study on bio-banding in male youth ice hockey: players' perceptions and coaches' selection preferences

Oliver Lindholm [1], Erik Niklasson [1, 2], John Lind [3], Daniele A. Cardinale [4,5], Tommy R. Lundberg [1,6]

**Affiliations**

1) Division of Clinical Physiology, Department of Laboratory Medicine, Karolinska Institutet, Stockholm, Sweden

2) Centre for Physical Activity Research, Copenhagen University Hospital - Rigshospitalet, Copenhagen, Denmark

3) Swedish Ice Hockey Association, Stockholm, Sweden

4) Department of Physiology, Nutrition and Biomechanics, The Swedish School of Sport and Health Sciences (GIH), Sweden

5) The Swedish Sports Confederation (Riksidrottsförbundet), Stockholm, Sweden

6) Unit of Clinical Physiology, Karolinska University Hospital, Stockholm, Sweden

**Corresponding author**

Tommy R. Lundberg, PhD

Email: tommy.lundberg@ki.se

Department of Laboratory Medicine

Division of Clinical Physiology, ANA Futura

Karolinska Institutet

14152 Huddinge, Sweden

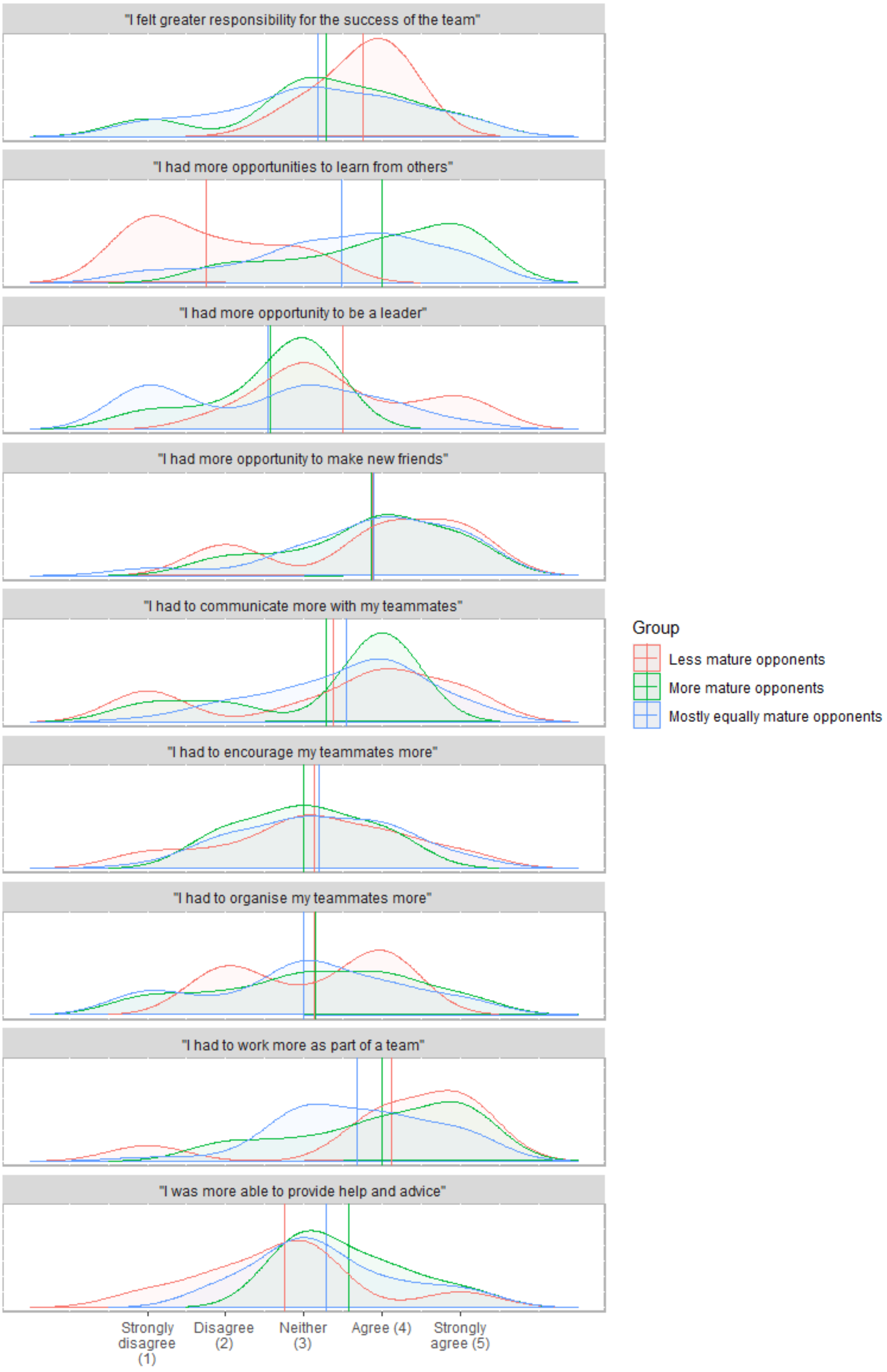


**Figure S1. Social aspects of Likert scale player perception in bio-bands compared to age-bands.** Density plots of Likert-scale responses. Statements are shown above the respective density plot. Less mature opponents: players assigned to U14 and BBL, More mature opponents: players assigned to U13 and BBH, Mostly equally mature opponents: players assigned to U13 and BBL or U14 and BBH. Vertical line: mean for each group.

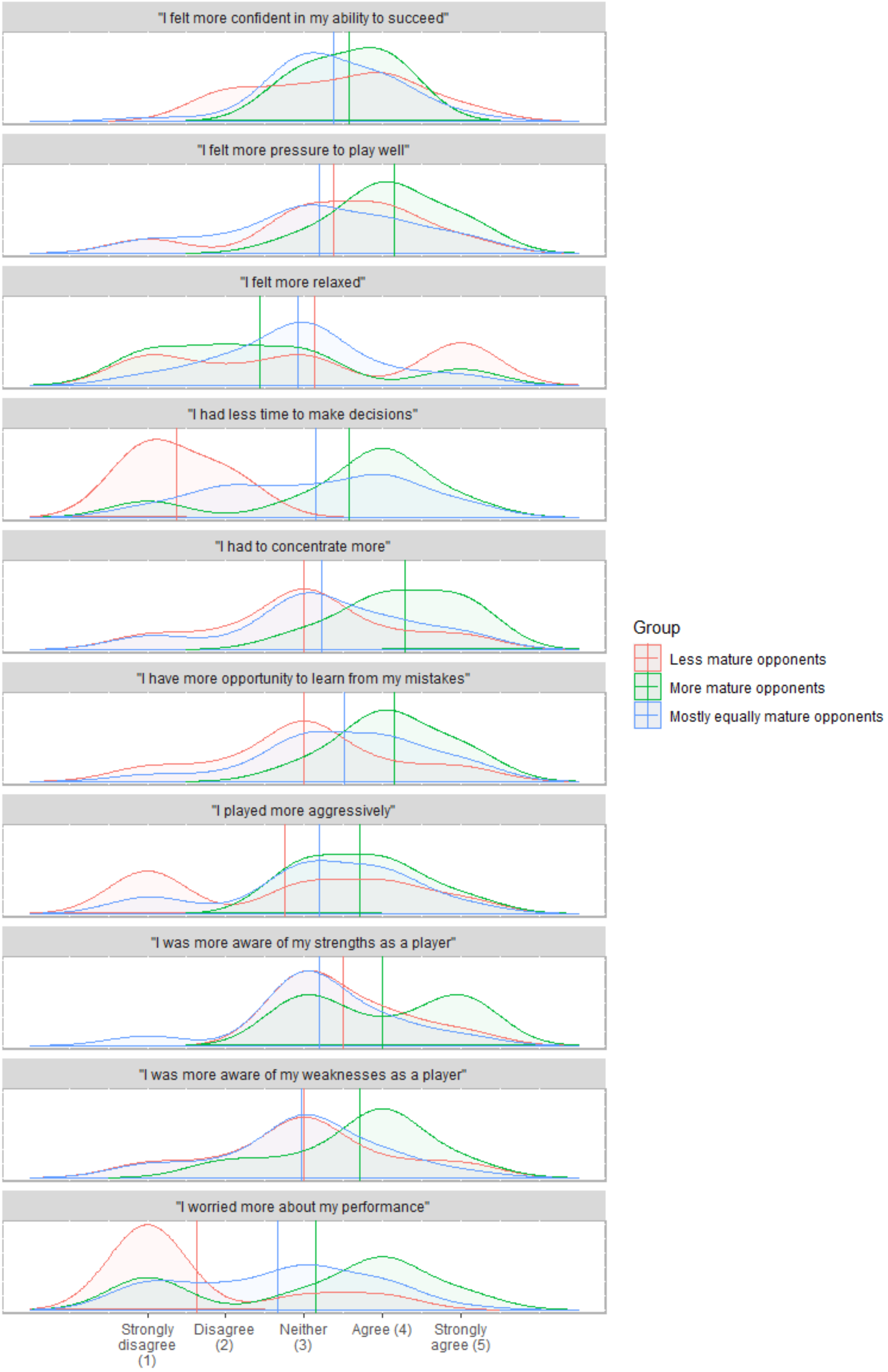


**Figure S2. Psychological aspects of Likert scale player perception in bio-bands compared to age-bands.** Density plots of Likert-scale responses. Statements are shown above the respective density plot. Less mature opponents: players assigned to U14 and BBL, More mature opponents: players assigned to U13 and BBH, Mostly equally mature opponents: players assigned to U13 and BBL or U14 and BBH. Vertical line: mean for each group.

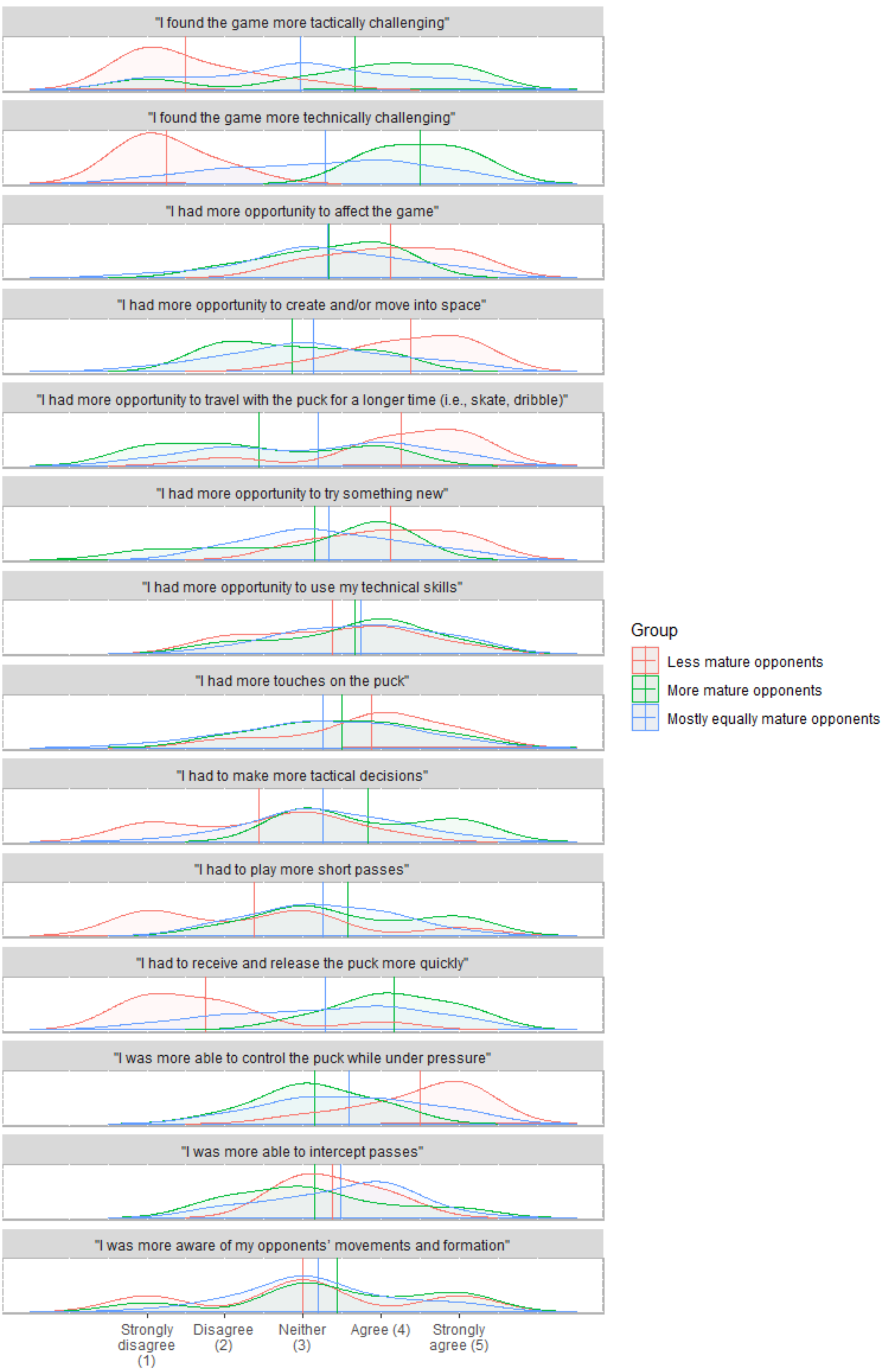


**Figure S3. Technical and tactical aspects of Likert scale player perception in bio-bands compared to age-bands.** Density plots of Likert-scale responses. Statements are shown above the respective density plot. Less mature opponents: players assigned to U14 and BBL, More mature opponents: players assigned to U13 and BBH, Mostly equally mature opponents: players assigned to U13 and BBL or U14 and BBH. Vertical line: mean for each group.

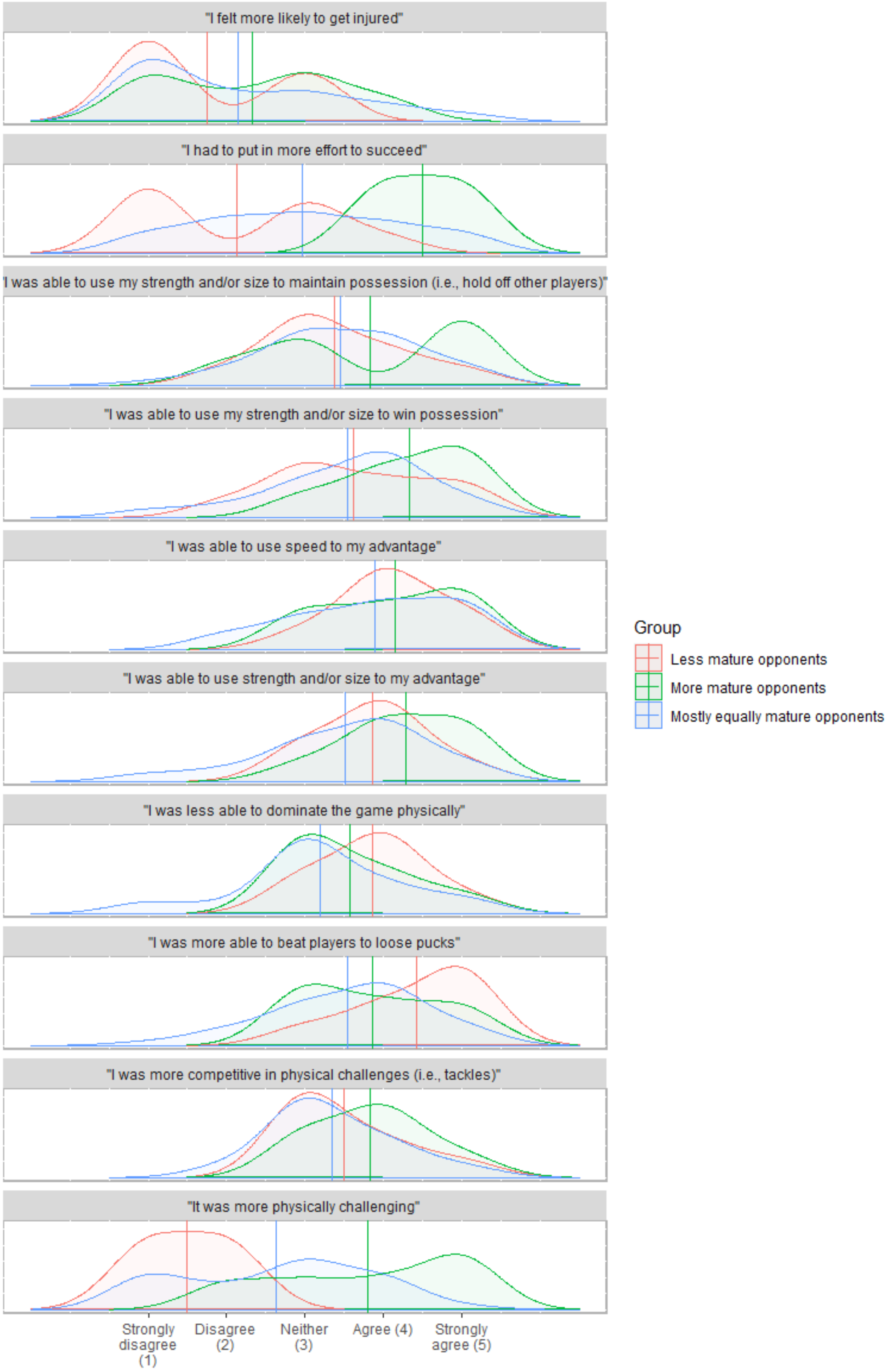


**Figure S4. Physical aspects of Likert scale player perception in bio-bands compared to age-bands.** Density plots of Likert-scale responses. Statements are shown above the respective density plot. Less mature opponents: players assigned to U14 and BBL, More mature opponents: players assigned to U13 and BBH, Mostly equally mature opponents: players assigned to U13 and BBL or U14 and BBH. Vertical line: mean for each group.

**Table S1.** Supporting data for Figure 6.

| Type of Match | Variable | R-value | P-value |
|---|---|---|---|
| BB | %PAH | -0.067 | 0.62 |
| AB | %PAH | -0.17 | 0.21 |
| BB | DA | 0.28 | 0.034 |
| AB | DA | 0.09 | 0.5 |
| BB | Z-score | -0.36 | 0.0056 |
| AB | Z-score | -0.28 | 0.029 |
| BB | Height | -0.085 | 0.53 |
| AB | Height | -0.19 | 0.15 |
| BB | Weight | -0.24 | 0.071 |
| AB | Weight | -0.32 | 0.015 |
| BB | HGS max | 0.25 | 0.082 |
| AB | HGS max | 0.17 | 0.23 |
| BB | PAH | -0.16 | 0.23 |
| AB | PAH | -0.18 | 0.18 |
| BB | SJ max | 0.24 | 0.091 |
| AB | SJ max | 0.20 | 0.16 |
| BB | SJ peak power | 0.013 | 0.93 |
| AB | SJ peak power | -0.13 | 0.38 |
| BB | SJ relative peak power (watt/kg) | 0.18 | 0.2 |
| AB | SJ relative peak power (watt/kg) | 0.11 | 0.44 |
| BB | YYIRT | 0.48 | 0.00048 |
| AB | YYIRT | 0.45 | 0.00098 |
| BB | 30 m sprint | -0.37 | 0.0089 |
| AB | 30 m sprint | -0.25 | 0.086 |

%PAH: percentage of predicted adult height, DA: Decimal age, HGS: Hand-grip strength, PAH: predicted adult height, SJ: squat jump, YYIRT: YoYo Intermittent Recovery Test level 1. AB: age-bands, BB: bio-bands.
