# Supplementary material for: A pilot study on bio-banding in male youth ice hockey: Players’ perceptions and coaches’ selection preferences
Source: PLoS One. 2024 Aug 12;19(8):e0308676. doi: 10.1371/journal.pone.0308676 (PMC11318913; doi:10.1371/journal.pone.0308676)
Supplement: Attachment — Submitted filename: Response to Reviewers.docx [file pone.0308676.s003.docx]

**RE: PONE-D-24-20888 A pilot study on bio-banding in male youth ice hockey: players' perceptions and coaches' selection preferences**

Dear editor,

We would like to thank the reviewers for their positive and constructive feedback. Please see below the point-by-point responses, along with the revised manuscript, which has improved. We hope that it now qualifies for acceptance.

Sincerely,

Tommy Lundberg with co-authors

**Journal requirement comments 1-5:**

Response: These have all been addressed.

**Reviewer #1:**

I would like to thank you for the opportunity to review this study, which is well-structured and of great value for the development of sports and youth training. Overall, the study is well-organized, involving significant logistics that underscore its importance. The only drawback I find is the lack of a greater difference in maturation, which would have allowed for a clearer distinction of the maturation influence. However, this is probably the scenario most frequently encountered in sports with these age groups. Therefore, I congratulate the authors on their work and offer a few suggestions that might be considered for improving the document.

Response: Thank you for this positive feedback. We agree with the drawback that only two age groups at the beginning of the growth spurt were studied; however, we are currently preparing an additional study on older age groups.

Introduction
Third paragraph – Please consider including the effects of maturation on the physical demands pointed out, i.e., not also the performance changes but what leads to that (which changes in Central Nervous System, metabolic changes…)

Response: Thank you for highlighting this, we have added this to the introduction. See revised version.

Line 70 to 74 – which is in line with other sports. Please consider strengthened this idea, including some references from other sports.
Response: Thank you for highlighting this, we have strengthen this idea as suggested.

Line 85 – in males
Response: We have added ”in males” in the text.

Line 86 – please consider including “in this pilot study”, otherwise it seems that you are refereeing to other study.
Response: We have replaced ”a” with ”this” in the text.

Methods
Including a subtopic of “intervention” seems that it is a longitudinal study. I suggest changing to something like “Test application” or other expression.
Response: Thank you for highlighting this, we have replaced ”intervention” with ”Bio-banding”.

Player perceptions – which is the questionnaire used? Is validated?
Response: Although the questionnaire has not been “validated” per se, it has previously been used in soccer and we refer to this study in the text. To our knowledge, this is the most reliable questionnaire in currently available for this type of study. At this stage, we are not sure how proper validation can be carried out effectively.

Discussion
For a better understanding of the results, maybe this session could be divided in subtopics as players and coaches’ point of view, discussing the physical characteristics (all tests performed), maturation (AB and BB) and perception/selection.
Response: Thank you for your suggestion. We have added subheadings in the discussion.

Limitations
Although very implemented, the Khamis-Roche analysis was not validated in Sweden. Therefore, it should be included in the limitation of the study these issues, indicating that some errors could be included as a result of differences in anthropometric nationalities.
Response: Thank you for highlighting this issue, we have added this to the limitation section.

**Reviewer #2:**

This is a novel and interesting study that explores a relatively new concept and strategy for the development of young athletes. The study is well designed and unique in nature and it is clear that the authors have a sound understanding of both growth, maturation, its assessment and the practice of bio-banding. So many papers that I read on this subject of growth and maturation are very ill informed, so it is refreshing to have the opportunity to read an informed paper. The methods of analysis are sound and the presentation of the results is excellent. I have some minor suggestions but in general think this is an excellent submission that should be prioritised for publication.

Response: Thank you for this positive feedback.

P2. L35. The R value should be r and there is no need to include the 0 just .57 as in r=.57
Response: This has been edited.

P3, L55. I am not sure prerequisites is the best word to use here. I assume you mean that these athletes possess substantial differences in size and athleticism and therefore may require different challenges in order to develop optimally?

Response: Thank you for this feedback. The statement has been revised.

P3, L61. Bio-banding is more of a strategy than a framework
Response: Thank you for highlighting this, we have replaced ”framework” with ”strategy”.

P3, L65. Premier League requires capitalisation
Response: Thank you for highlighting this, in the updated manuscript we use capitalisation for ”Premier League”.

P3, L70. More mature not matured…..may also say athletic than fit
Response: Thank you for highlighting this, we have changed ”matured” to ”mature” and ”fit” to ”athletic”.

P4, L96. I assume this was the biological parents who were assessed or self-reported height please clarify
Response: Thank you for this comment. We have revised the sentence for better clarity.

P6, L124. I assume the %PAH was compared against sex specific means and sds for children of the same age and sex? Please clarify.
Response: Thank you for this comment. We have revised the statement for better clarity in this regard.

P9, L25. No 0 values required for p values i.e., .13 not 0.13
Response: This has been revised.

P10, L225. I really like the graphs in figure 4, well done.
Response: Thank you very much!

P11, L236. The info in Figure 5 does not look like the items responses on the likert scale. I think you have made an error here.
Response: Thank you for highlighting this error, it’s been corrected.

P11, L242. I would assume the older players in the BB games would also be likely to be more delayed in maturity for their ages than the younger players in the same format. This would support the bio-banding concept that later developers are perceived more favorably in such contexts.
Response: Yes, that is correct. We have revised the paragraph accordingly.

P12, L272. Or maybe that coaches select players of varying maturity timing to certain positions?
Response: Yes, this could be the case. We cannot say for sure. All we can say is that early maturation increases probability of having a defensive position. We have revised the statement slightly to better reflect both possibilities.

P14, L322. It is equally plausible that the late maturing have to possess these abilities to survive rather than develop them. In fact some of the evidence would suggest the superior abilities of late developing soccer players is more likely to result from selection than challenge. Likelihood is both contribute but selection is more important.
Response: This may well be the case in general, although there was no formal selection process in these teams prior to this study, despite being an elite club. Nevertheless, we have revised the statement to allow for this possibility as well.
